# Incidence of HIV-Associated Tuberculosis among Individuals Taking Combination Antiretroviral Therapy: A Systematic Review and Meta-Analysis

**DOI:** 10.1371/journal.pone.0111209

**Published:** 2014-11-13

**Authors:** Tendesayi Kufa, Tonderai Mabuto, Evans Muchiri, Salome Charalambous, Dominique Rosillon, Gavin Churchyard, Rebecca C. Harris

**Affiliations:** 1 The Aurum Institute, Johannesburg, South Africa; 2 The School of Public Health, University of the Witwatersrand, Johannesburg, South Africa; 3 GlaxoSmithKline Vaccines, Wavre, Belgium; 4 CROMSOURCE on behalf of GlaxoSmithKline Vaccines, Wavre, Belgium; Institute of Infectious Diseases and Molecular Medicine, South Africa

## Abstract

**Background:**

Knowledge of tuberculosis incidence and associated factors is required for the development and evaluation of strategies to reduce the burden of HIV-associated tuberculosis.

**Methods:**

Systematic literature review and meta-analysis of tuberculosis incidence rates among HIV-infected individuals taking combination antiretroviral therapy.

**Results:**

From PubMed, EMBASE and Global Index Medicus databases, 42 papers describing 43 cohorts (32 from high/intermediate and 11 from low tuberculosis burden settings) were included in the qualitative review and 33 in the quantitative review. Cohorts from high/intermediate burden settings were smaller in size, had lower median CD4 cell counts at study entry and fewer person-years of follow up. Tuberculosis incidence rates were higher in studies from Sub-Saharan Africa and from World Bank low/middle income countries. Tuberculosis incidence rates decreased with increasing CD4 count at study entry and duration on combination antiretroviral therapy. Summary estimates of tuberculosis incidence among individuals on combination antiretroviral therapy were higher for cohorts from high/intermediate burden settings compared to those from the low tuberculosis burden settings (4.17 per 100 person-years [95% Confidence Interval (CI) 3.39–5.14 per 100 person-years] vs. 0.4 per 100 person-years [95% CI 0.23–0.69 per 100 person-years]) with significant heterogeneity observed between the studies.

**Conclusions:**

Tuberculosis incidence rates were high among individuals on combination antiretroviral therapy in high/intermediate burden settings. Interventions to prevent tuberculosis in this population should address geographical, socioeconomic and individual factors such as low CD4 counts and prior history of tuberculosis.

## Introduction

Human immune deficiency virus (HIV)-associated tuberculosis (TB) is an important public health problem particularly in high HIV prevalence settings. In 2012, the World Health Organisation (WHO) estimated that up to 1.1 million reported TB cases and 320 000 deaths from TB occurred in people living with HIV [1]. In the same year, up to 75% of all HIV-associated TB cases occurred in Sub-Saharan Africa [1]. Combination antiretroviral therapy (cART) or highly active antiretroviral therapy (HAART) reduces the risk of TB by 67% (95% CI 61–73%) among people living with HIV [2]. The risk of TB declines in proportion to the increases in CD4 counts after cART initiation [3]. In the high burden setting of Cape Town, South Africa, the risk of TB while on cART with a CD4 count of >700 cells/ml^3^ remained four fold higher than in HIV-uninfected persons from the same community [4]. Because cART alone is not sufficient to prevent HIV-associated TB, additional strategies are required. In order to develop additional strategies for preventing HIV-associated TB in people taking cART such as novel TB vaccines, an understanding of the incidence of and risk factors for HIV-associated TB in high/intermediate and low TB burden settings is required. We conducted a systematic review and meta-analysis to summarise and describe trends in the incidence of TB among adults taking cART in high/intermediate and low TB burden settings, stratified by geographical region, CD4 count, previous history of TB and duration on cART. We highlight the disparities in TB incidence rates between high and low TB burden settings and discuss the implications for interventions to further reduce the risk of HIV-associated TB among individuals on cART.

## Methods

### Search strategy and selection of papers

PubMed, EMBASE and Global Index Medicus databases were searched in parallel using search strings adapted to the requirements of each database (Table S1). For the PubMed search we conducted two separate searches using MeSH terms i) *“tuberculosis”* AND *“incidence”* ii) “tuberculosis” AND “HAART” with all the available qualifiers. Both PubMed searches were limited to papers describing studies in humans, published in English between 1^st^ January 2000 and 31^st^ March 2012. For the EMBASE search we used EMTREE terms “tuberculosis” AND “incidence” OR “HAART” with all the available qualifiers and limited the search to papers describing studies in humans, published in English between 1^st^ January 2000 and 31^st^ March 2012. For the Global Index Medicus search, we searched all indexes and all sources (which include AIM, LILACS, IMEMR, IMSEAR, WPRIM, WHOLIS and Medline) using the keywords *“tuberculosis” “incidence” “HAART”* and limited the search to studies written in the English language. No other limits applied.

The search outputs were imported into a combined file in reference management software and duplicates removed. Two independent reviewers (TM and TK) screened all titles and abstracts to identify papers for full text review. Full texts were then screened by the same reviewers and eligibility criteria applied. Eligibility for inclusion required reporting a TB incidence rate for a cohort of individuals on cART and more than 100 participants included in the cohort. Review papers, papers exclusively reporting multi-drug or extensively drug resistant (MDR/XDR) TB as outcomes, and papers reporting exclusively on children younger than 15 years of age were not eligible. Where discordance occurred in the independent review of papers, the papers were discussed and consensus achieved. References lists included in the eligible papers were hand searched in order to identify additional eligible papers. The search criteria and other methods used in the review were included in a protocol outline agreed upon by the authors before data collection commenced. This protocol outline can be found in the supplementary information (Table S2).

### Abstraction of data from eligible papers

Data on study characteristics (including but not limited to: author, date of publication, location, sample size, cohort characteristics and TB incidence rates) were abstracted using a standardized form (Table S3). If a study described two or more distinct cohorts, data was abstracted for each of the cohorts meeting the criteria for inclusion in the review.

Estimates of national TB incidence rates, national adult HIV prevalence rates and World Bank income classification for the country and year each study was published were obtained from the WHO database of TB burden [5], UNAIDS Global HIV estimates [6], [7] and the World Bank classifications respectively [8]. We used the estimates of national HIV prevalence rates last updated in 2009, and the estimates of national TB incidence rates last updated in 2010. Therefore, the HIV estimates from 2009 were used for studies published after 2009 and, the 2010 estimates of national TB incidence were used for studies published after 2010. For multi-country studies, the data on national TB incidence and HIV prevalence rates were not abstracted but were assigned to low or high burden depending on the average national TB incidence rate in the countries the cohorts were from. Cohorts described in the studies were classified as high/intermediate burden if the estimated national TB incidence rate for the country and year were ≥25 per 100 000 population per year, and as low burden if the TB incidence rates were below this threshold. Cohorts were also classified as being from low, middle or high income settings according to the World Bank income classification.

### Assessment of study quality

Study quality was assessed using a standardized tool (see Table S4) adapted from the Newcastle-Ottawa Scale (NOS) for cohort studies [9]. The tool was used to assess the following study characteristics: sampling methods, presence of sampling bias, exclusion of TB at cohort entry, outcomes ascertainment during follow up, duration of follow up and loss to follow up rates. Each of these criteria was assigned a score as shown in Box 1 (Table S4). The highest possible score was 6 and studies with scores ≥4 were considered to be of good quality. No studies were excluded from the reviews on the basis of their quality scores.

### Data analysis and presentation

In the qualitative part of the review, all eligible cohorts reporting a TB incidence rate among individuals on cART were classified into high/intermediate and low burden settings and described with respect to cohort characteristics. TB incidence rates were summarized according to CD4 cell count strata, duration on cART and by prior history of TB. Where studies only reported number of TB cases and person-years of follow up for the different strata in the cohort, the incidence rates and confidence intervals were computed in Stata 12 (Stata Corporation, College Station, Texas, USA).

### Meta-analysis

Meta-analyses (quantitative reviews) were conducted to determine summary estimates of the TB incidence rates among HIV-infected individuals on cART overall and point estimates across different categories or strata of study quality, study designs, national HIV prevalence rates, national TB incidence rates, CD4 count, durations on cART and prior history of TB. To be eligible for inclusion in meta-analyses, studies were required to report both the number of TB cases and person-years of follow up by the different categories listed. These fields were required to enable estimation of incidence rates of TB and associated standard errors using the random effects model. These data were recorded onto the abstraction forms and entered into an Excel 2007 sheet (Microsoft Corporation, Washington, USA) and exported into Stata 12 for analysis. I-squared estimates were used to determine heterogeneity between studies.

## Results

### Summary of studies

**Figure 1:**
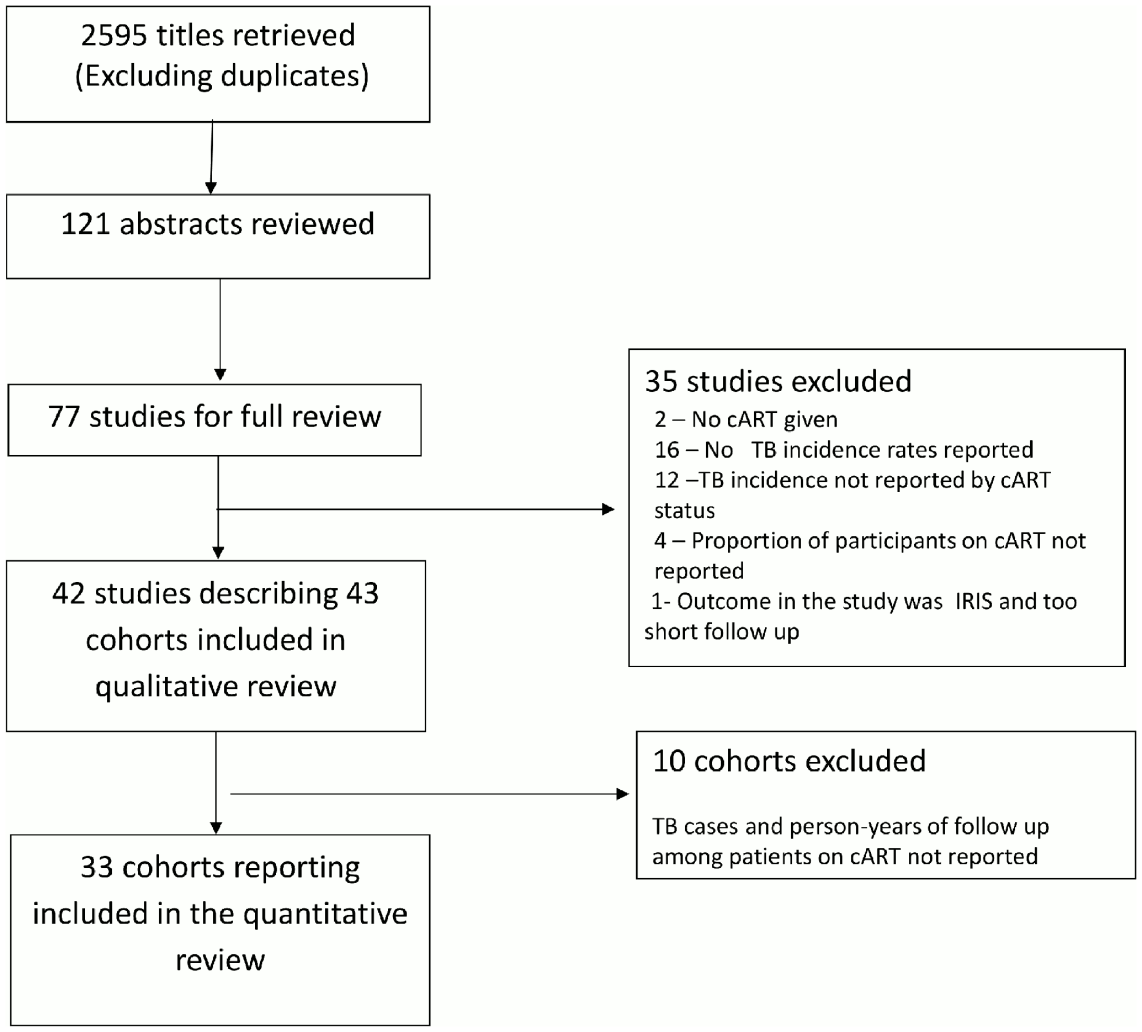
Summary of search findings.

From 2945 unique study titles retrieved, 121 titles were eligible for abstract review and 77 for full text review. From the full texts reviewed, 42 studies [4], [10]–[50] describing 43 cohorts were eligible for inclusion in the qualitative review and a subset of 33 cohorts for inclusion in the quantitative review (meta-analysis - Figure 1). Of the 43 cohorts, 32 (74%) were from high/intermediate burden settings with national TB incidence rates ranging from of 46 to 981 per 100 000 and national HIV prevalence rate ranging from 0.3% to 18.2%. Eleven cohorts (26%) were from low burden settings with national TB incidence rates ranging from 4.1 to 17 per 100 000 population per year and national HIV prevalence rate of 0.2% to 0.6%. The full list and characteristics of papers included in the review are presented in Table S5.

Characteristics of cohorts from high/intermediate and low burden settings are presented in Table 1. When compared to cohorts from low burden settings, cohorts from high/intermediate burden were smaller in size, had lower median CD4 cell counts at study entry and had fewer person-years follow up. TST positivity was reported for a few cohorts in both settings (five cohorts (15.6%) from high/intermediate burden settings and five (45.5%) cohorts from low burden settings).

**Table 1:** Characteristics of study cohorts from high burden settings and from low burden settings.

Characteristic	High/intermediate burden (N = 32)		Low burden (N = 11)	
	number of cohorts		number of cohorts	
Prospective study design (n, %)	32	16 (50)	11	2 (18.2)
Cohort size (range)	32	101–19325	11	1824–72850
Cohorts from clinical care settings (n, %)	32	24 (75)	11	11 (100)
% males in the cohort, (range)	31	21–91	10	67–77
Median/mean age of cohort, (range)	28	31–38	10	33–41
% of cohort with prior history of TB, (range)	20	0–100	3	0–13.9
% of cohort who were MSM, (range)	4	18.7–59	6	17.3–58.7
% of cohort who were IDU, (range)	3	1.6–14	6	4.8–59
% of cohort on cART at baseline/by end of study, (range)	32	5.7–100	11	10.2–100
Cohort with 100% of participants on cART, (n, %)	32	22 (68.8)	11	5 (45.5)
% of cohort on NNRTI-based treatment, (range)	10	35–100	2	26.3–33
% of cohort on PI- based treatment, (range)	5	3.2–73	2	56–63
Median CD4 count at cohort entry, cells/µl), (range)	27	37–444	10	207–422
Median CD4 count at cART initiation(cells/µl), (range)	20	97–252	5	207–297
Median duration of follow up in months, (range)	24	8–73	4	15.8–56.4
Person-years of follow up, (range)	27	159–18162	10	14711–427957
Person-years of follow up on cART,(range)	25	65.1–18162	8	11248–313807
Availability of mycobacterial culture, (n, %)	29	19 (65.3)	10	10 (100%)

N is total number of cohorts, n is number of cohorts with the characteristic, IQR – interquartile range, MSM- men who have sex with men, IDU- intravenous drug user, NNRTI – non-nucleoside reverse transcriptase inhibitors, PI – protease inhibitors, cART- combination antiretroviral therapy.

### Study quality

Table 2 describes the findings of the study quality assessment. The overall quality scores ranged from 2–6 with the majority of the papers (27 of 42 studies, 64%) being considered to be of acceptable quality (score ≥4).

**Table 2:** Summary of study quality.

Criteria	high burden (N = 32)		low burden(N = 11)	
	n (%)	studies	n (%)	studies
*1. Sampling method*				
Reported sampling method used	32 (100)	all studies	11 (100)	all studies
*2. Sampling bias*				
Assessed and reported on sampling bias	18 (56.3)	4,11, 14, 17, 19, 20, 22, 24, 30, 31,	2 (18.2)	29, 32
		33, 34, 36,37, 38, 39, 43,47		
*3. Screening of TB at study commencement*				
Reported screening/exclusion of TB at cohort entry	32 (100)	all studies	11 (100)	all studies
*4. Ascertainment of outcomes*				
Smear + culture + clinical + chest radiograph	21 (62.5)	4, 14, 17, 19, 20, 21, 24, 27, 28, 30,	7 (63.6)	12, 15, 26, 32, 35, 48, 49
		31, 33,34, 35, 36, 37, 42, 43,46, 47,		
		49		
Smear + clinical + chest radiograph (no culture)	7 (21.2)	10, 11, 25,38, 39, 41, 50		
Clinical + chest radiograph (no smear or culture)	1 (3.0)	30	0 (0)	
Treatment initiation records only	2 (6.1)	22, 23,	1 (9.1)	18
Not specified	2 (6.1)	40, 45	3 (27.2)	16, 29, 44
*5. Median duration of follow up*				
Median follow-up <9 months	1 (3)	41	0 (0)	
Median follow-up ≥9 months	23 (72.7)	4, 14, 17, 19, 20, 21, 22, 23, 24, 25	5 (45.4)	15, 18, 29, 32,44
		28, 30, 31, 33, 34, 36, 37,39, 42,		
		43, 45, 46, 47		
Median follow up not reported	8 (24.2)	10, 11, 27, 35, 38, 40, 49, 50	6 (55.5)	12, 13, 16, 26, 48, 49
*6. Loss to follow up*				
<20% of participants lost to follow-up	15 (45.5)	11,14, 17, 19, 20, 21, 22, 23, 24, 27	1 (9.1)	29
		28, 31, 36, 39, 40		
>20% of participants lost to follow-up	1 (3.0)	4	0	
Loss to follow up not reported	16 (48.5)	10, 25, 30, 33, 34, 35, 37, 38, 41,	10 (90.9)	12, 13, 15, 16, 18, 26, 32, 44,
		42, 43, 45, 46, 47, 49, 50		48, 49
*Overall study quality score (median, range)*	5 (2–6)		3 (2–5)	
*Studies with quality score ≥4*	24 (72.7)	4, 11, 14, 17, 19, 20, 21, 22, 23, 24,	3 (27.3)	15, 29, 32
		27, 28, 30, 31, 33, 34, 36, 37,38,		
		39, 42, 43, 46, 47,		

n = number of studies with characteristic.

### Qualitative review of TB incidence rates

Table 3 summarises the characteristics of TB cases and the TB incidence rates reported across different CD4 count and duration on cART strata, for both high/intermediate and low burden cohorts. The proportion of TB cases with pulmonary TB was higher in cohorts from high/intermediate burden settings compared to those from low burden settings. The median CD4 counts at study entry among individuals who subsequently developed TB were similar between high/intermediate and low burden cohorts. The TB incidence rates reported among individuals on cART were seven to 30 times higher in cohorts from high/intermediate burden settings compared to those from low TB burden settings.

**Table 3:** TB cases characteristics and incidence rates reported in high burden cohorts and in low burden cohorts.

Cohort characteristics	High/intermediate TB burden		Low TB burden	
	(N = 32)		(N = 11)	
	Number of cohorts reporting		Number of cohorts reporting	
Proportion (%) of TB cases with pulmonary TB (range)	16	43–81	6	44–65
Proportion (%) of TB cases who are male (range)	5	33.2–67.6	4	63.3–91
Median CD4 count of TB cases at study entry, cells/µl (range)	8	75–197	3	80–179
TB incidence rate among those on cART,	32	0.6–10.5	11	0.02–1.5
cases/100person-years, (range)				
TB incidences across baseline CD4 count strata				
cases/100person-years, (range)				
<100	6	0.6–6.8	1	0.84
101–200	2	1.7–4.8	0	.
201–350	3	1.7–3.7	1	0.47
351–500	3	1.8–3	1	0.19
>500	1	2	1	0.17
TB incidences across current CD4 count strata,				
cases/100person-years,(range)				
<100	4	8.9–25.5	3	0.11–0.83
101–200	4	3.6–11.2	1	0.09
201–350	3	1.8–7.8	2	0.08–0.26
351–500	3	0.7–5.0	3	0.04–0.21
>500	2	1.5–4.1	2	0.06–0.1
TB incidences with increasing duration on cART				
cases/100person-years,(range)				
0–3months	14	3.4–23	5	0.22–1.7
3–6months	10	2.2–10.7	4	0.15–1
6–12months	10	1.2–7.0	5	0.07–0.62
12–24 months	13	1.3–6.7	3	0.07–0.33
24–36 months	7	1.4–7.4	3	0.09–0.18
>36 months	4	0.4–5.8	1	0.05
TB incidence among those with prior history of TB,	6	1.9–11.9	1	1.19
cases/100person-years, (range)				
TB incidence among those with no prior history of TB	6	1.8–8.1	1	1.83
cases/100person-years, (range)				

N is total number of cohorts, n is number of cohorts with the characteristic, IQR – interquartile range, cART- combination antiretroviral therapy.

In cohorts from high/intermediate and low burden settings, TB incidence rates generally increased with decreasing current CD4 counts and CD4 count at cART initiation, more so with CD4 counts less than 200 cells/µl (Table 3). TB incidence rates also increased with durations on cART less than six months. In six cohorts from high/intermediate burden settings, TB incidence rates were higher among those with prior history of TB compared to those with no prior history of TB (1.9–11.9 per 100 person-years compared to 1.8–8.1 per 100 person-years).

TB incidence rates among individuals on cART also varied with geographical location with highest incidence rates found in cohorts from Sub-Saharan Africa (range 0.9–7.82 per 100 person-years, n = 23), followed by those in Asia (range 1.32–2.83 per 100 person-years, n = 2), in South America (0.2–2.6 per 100 person-years, n = 4), and in Europe and North America (range 0.02–1.9 per 100 person-years, n = 9). Rates were much higher among cohorts from low income countries (range 0.9–8.6 per 100 person-years, n = 16) and middle income countries (0.6–10.5 per 100 person-years, n = 16); compared to those from high income countries (0.02–1.9 per 100 person-years, n = 9).

### Meta-analysis of TB incidence among HIV-infected adults on cART

**Figure 2:**
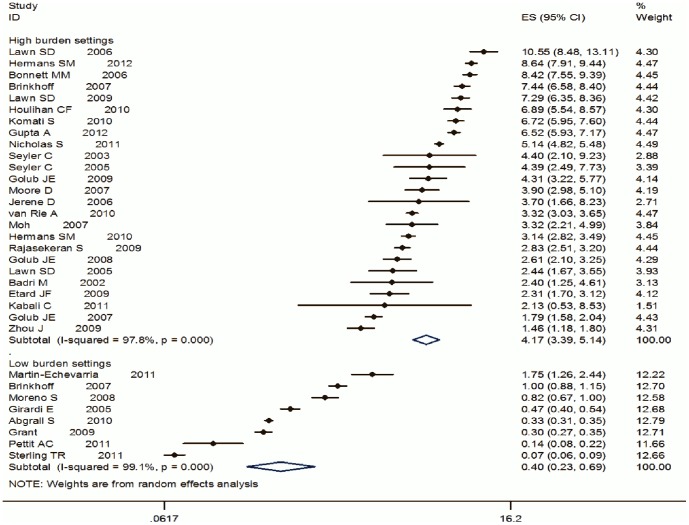
Forest plot showing summary estimates of TB incidence rates among individuals on cART comparing cohorts from high TB burden settings to low TB burden settings.

Thirty-three cohorts were eligible for inclusion in the meta-analysis. (See Figure 2). Heterogeneity was computed separately for high/intermediate (I^2^ = 98%, p-value <0.001) and low (I^2^ = 99.1%, p-value <0.001) burden settings and was large in both settings. As expected, the summary estimate of TB incidence among those on cART was higher for cohorts from high/intermediate burden settings compared to those from the low burden settings–4.17 per 100 person-years (95% CI 3.39–5.14 per 100 person-years) vs. 0.4 per 100 person-years (95% CI 0.23–0.69 per 100 person-years, (Figure 2). In the analyses stratifying summary estimates of TB incidence rates by study quality, study design (retrospective or prospective studies), national TB incidence rates and national HIV prevalence rates (see Table 4), heterogeneity remained high. This implied that these variables did not explain most of the heterogeneity observed in the TB incidence rates.

**Table 4:** Summary estimates from meta-analysis of TB incidence rates stratified study level variables.

Variable		High/intermediate burden			Low burden			
	N Studies	Summary estimate (per 100 person-years)	I^2¥^	p^*^	N studies	Summary estimate (per 100 person-years)	I^2^	p^*^
**Study quality**								
low	10	3.7 (2.3–5.6)	98.8%	<0.001	6	0.37 (0.18–0.77)	93.3%	<0.001
high	14	4.5 (3.6–5.5)	95.5%	<0.001	2	0.50 (0.19–1.31)	98.4%	<0.001
**Study Design**								
Retrospective	13	3.7 (2.7–5.3)	98.7%	<0.001	6	0.40 (0.16–1.04)	99.3%	<0.01
Prospective	12	4.6 (3.6–6.0)	95.2%	<0.001	2	0.39 (0.28–0.55)	943%	<0.01
**National TB incidence**								
**rates (per 100 000**								
**population)**								
<25					6	0.34 (0.18–0.64)	99%	<0.001
25–200	7	2.8 (2.1–3.6)	83.7%	<0.001				
201–800	6	3.7 (2.1–6.4)	98%	<0.001				
>800	8	5.6 (4.2–7.4)	96.7%	<0.001				
**National HIV prevalence**								
**rates**								
<1%	4	2.3 (1.8–3.0)	88.8%	<0.001	6	0.34 (0.18–0.64)	99%	<0.001
1–4.9%	3	3.7 (2.7–4.9)	0	0.735				
5–10%	5	4.3 (2.3–8.1)	98.1%	<0.001				
>10%	9	5.2 (3.9–6.9)	96.3%	<0.001				

¥ I^2^- Amount of heterogeneity not explained by the variable, *p-value for heterogeneity not explained by the variable, N – number of cohorts.

### Meta-analysis of TB incidence among HIV-infected adults on cART stratified by CD4 counts, duration on cART and prior history of TB

Summary estimates of TB incidence rates stratified by CD4 counts at entry, duration on cART and prior history of TB are shown in Table 5. The summary estimates of the TB incidence rates were higher in cohorts from high/intermediate burden settings compared to those from low burden settings across all baseline CD4 count strata, duration on cART and prior history of TB strata, although inference was limited by number of cohorts. Among cohorts from high/intermediate burden settings, TB incidence rates were higher in the baseline CD4 count <200 cells/µl stratum compared to those in 200–350 cells/µl or >350 cells/µl strata. There was significant heterogeneity in the TB incidence rates across the different strata in the meta-analysis.

**Table 5:** Summary estimates from meta-analysis of TB incidence rates stratified by baseline CD4 count, duration on cART and previous history of TB.

Variable		High/intermediate burden				Low burden		
	N	Summary estimate	I^2¥^	p^*^	N	Summary estimate	I^2^	p^*^
	Studies	(per 100 person-years)			Studies	(per 100 person-years)		
**Baseline CD4 count**								
<200	5	4.47 (3.55–5.63)	89.6%	<0.001	1	0.84 (0.31–1.62)	-	-
200–350	3	2.32 (1.54–3.51)	42.3%	0.177	1	0.46 (0.35–0.60)	-	-
>350	3	2.34 (1.78–3.08)	0.00%	0.877	1	0.23 (0.16–0.34)	-	-
**Duration on cART**								
<3months	6	13.67 (10.62–17.60)	86.9%	<0.001	3	0.73 (0.27–1.99)	96.3%	<0.01
3–6 months	6	6.11 (4.62–8.09)	76.4%	0.001	3	0.46 (0.18–1.19)	93.6%	<0.01
6–12 months	6	3.14 (2.20–4.48)	83.9%	<0.001	3	0.29 (0.12–0.74)	94.5%	<0.01
12–24 months	4	3.94 (1.97–7.88)	94.5%	<0.001	3	0.19 (0.08–0.47)	94.8%	<0.01
>24 months	1	5.87(5.15–6.68)	-	-	2	0.09(0.03–0.23)	91.3%	<0.01
**Previous History TB**								
Yes	4	4.74 (2.10–10.73)	93.1%	<0.001	1	1.20 (0.39–3.71)	-	-
No	4	2.78 (1.36–5.68)	97.1%	<0.001	1	1.83 (1.30–2.59)	-	-

¥ I^2^- Amount of heterogeneity not explained by the variable, *p-value for heterogeneity not explained by the variable, N – number of cohorts.

## Discussion

This review summarises and describes trends in TB incidence rates among HIV-infected adults on cART, comparing cohorts from high/intermediate burden settings with those from low burden settings. In the qualitative review, the incidence rates in cohorts from high/intermediate burden settings were seven to 30 times higher than rates in cohorts those from low burden settings. In the quantitative review the rates in high/intermediate burden settings were 10 times higher than those from low burden settings. Rates were highest in cohorts from low/middle income countries, from Sub-Saharan Africa, especially among individuals with baseline and current CD4 counts less than 200 cells/µl and among those on cART for less than six months.

### Geographic and socio-economic factors

Our review suggests that background socio-economic conditions as measured by the World Bank classification may be associated with increased TB incidence among individuals on cART. In a 2009 study, Dye *et al* found that improvements in sanitation, lower child mortality and a higher human development index were associated with declines in national TB notification rates among 134 countries studied [51]. In this review, Sub-Saharan Africa, a region largely consisting of low income countries had the highest TB incidence rates among HIV-infected individuals on cART and should be a priority region for implementation of public health interventions which address social determinants of TB.

### Individual level factors

The high rates of TB among HIV-infected adults on cART in this review, particularly in cohorts from high burden settings confirm that cART alone is not sufficient to prevent TB in populations with HIV [2]. Results reported here indicate that additional strategies at individual level are required to prevent HIV-associated TB particularly for individuals who have low CD4 counts, previous history of TB disease or recently initiated cART. Low CD4 counts during treatment with cART may be a result of i) late presentation for cART initiation, ii) sub-optimal immune reconstitution, or iii) treatment failure on cART [52], [53]. Concerted efforts should be made to ensure initiation of cART at higher CD4 counts, better adherence to cART and better retention in HIV care. The high rates of TB in the first six months after cART initiation observed in this review may be attributed to inadequate TB screening at start of cART [52], or to unmasking of subclinical TB due to restoration of TB specific immune responses or paradoxical immune reconstitution syndrome (IRIS). [52]–[54] IRIS has been associated with low CD4 counts at cART initiation and could be avoided by initiating cART at higher CD4 counts, [52]–[54] or by adequately excluding and treating TB before initiating cART. In settings with both high HIV prevalence and high TB transmission, TB in individuals with HIV can be also prevented through the scaling up of TB preventive therapy and strengthening TB infection control measures at facility and community levels as repeated TB episodes among those on cART have been linked to re-infection [55].

### Implications for further research

The findings of this review can inform the development of additional tools - such as new TB vaccines and drug regimens for TB preventive therapy - to prevent TB in HIV-infected populations. Should a vaccine candidate or preventive therapy regimen progress to phase IIb or III trials including HIV-infected subjects and measuring TB disease as an endpoint, large numbers of subjects will be needed to obtain a sufficient number of endpoints in the trials. High/intermediate burden settings should be priority locations for the conduct of such trials to help minimise the required sample size. However, with cART initiation occurring at increasingly higher CD4 count thresholds and use of isoniazid preventive therapy (IPT) being scaled up in most settings, the expected number of endpoints in such HIV-infected populations may be smaller and such trials may become infeasible even in high/intermediate burden settings. Prioritising enrolments of participants with prior history of TB may increase the number of endpoints in such trials.

### Strengths and limitations

Summarising the TB incidence rates among individuals taking cART emphasises the magnitude of the TB burden especially in high TB and HIV burden settings. Such data highlight the limitations of cART as a tool for TB prevention in such settings, with challenges remaining due to ongoing transmission, late presentation into care or sub-optimal immune restoration in cART care. This review included 42 studies, the majority of which were of good quality. However this review also had some important limitations. We restricted our search to literature published in English as we did not have resources for translation. We did not search conference abstracts as these were likely to have incomplete follow up data. Twenty-three percent of the cohorts included in the qualitative review were not eligible for inclusion in the meta-analysis because they did not report number of TB cases, or the person – years of follow up stratified by use of cART. There was limited duration of follow up of participants especially in studies from high/intermediate burden settings. The association of longer duration of follow up in the cohort with reduction in TB incidence was evident in the reported rates and from the meta-analysis. This may have contributed to higher rates of TB observed in the cohorts from high/intermediate TB burden settings. There was large heterogeneity in the TB incidence rates in both high/intermediate burden and low TB burden settings which was not fully explained by study variables in stratified analyses. Conducting univariable or multivariable meta-regression analyses would have allowed us to better the determine factors accounting for the heterogeneity observed but the limited number of studies particularly from low TB burden settings precluded this. TB incidence rates in individuals on cART are likely influenced by local TB transmission and the rates of reactivation of latent infection. Data on TST positivity were not available for most studies and any heterogeneity as a result of these two variables could not be accounted for. Another limitation was that the review and meta-analysis included data collected at the individual level in the studies which were then aggregated to give study level variables. This made the review prone to aggregation bias.

Despite these limitations, the study provides valuable information for the evaluation, planning and implementation of preventive strategies for HIV-associated TB. Strategies to further reduce the risk of TB among individuals on cART such as the use of TB preventive therapy regimens, early initiation of cART, better TB screening at initiation of and during cART, and TB infection control can be appropriately targeted based on these data.

## Supporting Information

Table S1 **Summary of searches.**
(DOCX)

Table S2 **Protocol outline.**
(DOCX)

Table S3 **Data abstraction form.**
(DOCX)

Table S4 **Modified Ottawa Scale.**
(DOC)

Table S5 **List of studies included.**
(XLS)

Checklist S1 **PRISMA checklist.**
(DOC)
