# Supplementary material for: Incidence of HIV-Associated Tuberculosis among Individuals Taking Combination Antiretroviral Therapy: A Systematic Review and Meta-Analysis
Source: PLoS One. 2014 Nov 13;9(11):e111209. doi: 10.1371/journal.pone.0111209 (PMC4230893; doi:10.1371/journal.pone.0111209)
Supplement: Table S1 — Summary of searches. (DOCX) [file pone.0111209.s001.docx]

**Supplementary Appendix 1: Summary of search terms used**

| ***Database*** | ***PubMed*** | ***EMBASE*** | ***Global Health Library*** |
| --- | --- | --- | --- |
| *Search terms* | *Mesh terms:*  "Tuberculosis"[Mesh] AND "Incidence"[Mesh] AND ("humans"[MeSH Terms] AND English[lang]) AND ("2000/01/01"[PDAT] : "2012/03/31"[PDAT])  ("Antiretroviral Therapy, Highly Active"[Mesh] AND "Tuberculosis"[Mesh]) AND ("humans"[MeSH Terms] AND English[lang]) AND ("2000/01/01"[PDAT] : "2012/03/31"[PDAT]) | *EMTREE terms :*  'tuberculosis'/exp OR 'tuberculosis' AND ('incidence'/exp OR 'incidence' OR 'highly active antiretroviral therapy'/exp OR 'highly active antiretroviral therapy')  Limits applied: studies done in humans , published in English between 1^st^ January 2000 and 31^st^ March 2012 | Key word search: *tuberculosis incidence HAART* (no comma’s).  Limits applied - English language.  All indexes and all sources searched. |
| *unique hits* | *1567*  *449* | *1109* | *219 unique hits* |
| *Unique titles (excluding duplicates* | *2945 unique titles/ abstracts* | | |
| *Abstracts* | *121abstracts reviewed* | | |
| *Full text review* | *77 papers reviewed* | | |
| *included in systematic review* | *42 papers describing 43 cohorts* | | |
