# Supplementary material for: Incidence of HIV-Associated Tuberculosis among Individuals Taking Combination Antiretroviral Therapy: A Systematic Review and Meta-Analysis
Source: PLoS One. 2014 Nov 13;9(11):e111209. doi: 10.1371/journal.pone.0111209 (PMC4230893; doi:10.1371/journal.pone.0111209)
Supplement: Table S2 — Protocol outline. (DOCX) [file pone.0111209.s002.docx]

**Concept Note: Incidence of tuberculosis among HIV infected patients on combination ART**

| **Titles:** | The incidence of tuberculosis among HIV infected patients on cART : a systematic review of literature |
| --- | --- |
| **Project Lead:** | Tendesayi Kufa |
| **Collaborators:** | Edith Roset Bahmanyar, Suzanne Verver, Salome Charalambous, Gavin Churchyard |
| **Background** | TB remains the leading cause of death among HIV infected people. Globally 13% of all TB cases that occurred in 2009were among HIV infected individuals. Sub-Saharan Africa accounted for 80% of this burden of HIV associated TB cases. HIV may increase the risk of infection with TB following exposure and accelerates the progression of latent TB infection to disease with incidence of disease 20 times higher than HIV uninfected individuals. The interaction between TB and HIV on different levels makes it unlikely that DOTS alone will be sufficient to control TB will in high HIV prevalence settings. Additional interventions such preventive therapy and new TB vaccines will be required to control TB. The ideal TB vaccine will have to be effective and safe when administered to HIV infected individuals including those on ART. The vaccines need to be sufficiently immunogenic in order to provide some degree of protection from TB without triggering the progression of patient’s HIV disease. In order to identify appropriate end points for future phase III trials of TB vaccines, it is necessary to understand the epidemiology of TB disease in populations with mild to moderate immunosuppression as a result of HIV infection. In meta-analyses ART reduces the incidence of TB 67% (95% CI 61- 73%) regardless of tuberculin skin test status and mortality by 64- 95% among HIV infected individuals. Despite this the risk of TB remains at least two fold greater among HIV infected individuals compared to non-HIV infected. For the reason any novel TB vaccine would also be indicated for HIV infected on ART. This paper will review the incidence of tuberculosis among HIV infected patients on cART |
| **Objectives** | 1. Summarize studies describing the incidence of TB among HIV infected individuals on cART. 2. Describe the distribution of the TB incidence in this population across :   Different geographical regions/ burden of tuberculosis  Increasing duration on cART  Previous/ prior history of TB at initiation of cART  Different CD4 count strata at baseline/ cART initiation  Different current CD4 count stratum   1. Identify the strengths and weaknesses in these studies 2. Identify priority areas for further research 3. To identify and make recommendations for future vaccine trials, current practice and policy |
| **Methods** | *Study Design:* Systematic review of literature  *Search criteria*  A search of Medline, EMBase, CINAL databases will be done using the following key words :    **tuberculosis, incidence, ART, HAART**  In addition conference databases were searched and experts in the field contacted for additional publications.  *Inclusion and Exclusion criteria:*  In order to be included in the review studies have to:   - Include description of study design, methodology and statistical analysis - Include descriptions of incidence of TB disease among HIV infected individuals on ART - Studies conducted in both high burden and low burden TB countries - Have been published after January 1^st^ 2000 - Study population or sample size population greater than 100 - Reports more than 10 incident TB cases   Review articles and articles on MDR/XDR TB and will be excluded from analysis. Studies reporting outcomes in children less than the age of fifteen will also be excluded.  *Data extraction*  Abstracts and citations from publications retrieved from initial search will be stored into a database. The abstracts will be screened for eligibility. Articles of abstracts are considered eligible after initial screen will be retrieved and data extracted using a data abstraction form. The following data elements will be abstracted onto forms and entered into an Excel spreadsheet.   - **Date of publication** - **location (setting, country, region) where study was conducted** - **objectives of the study, population** - **study designs (prospective/ retrospective)** - **HIV prevalence rates in the population** - **TB notification rates/ estimated TB incidence rates in country where study was conducted** - **Proportion of study participants with use of IPT** - **proportion of participants with prior history of TB** - **proportion of participants who are male,** - **median baseline CD4 counts in study population,** - **duration of follow up** - **study quality** - **number of incident TB cases** |
| **Findings/ Results** | Findings of the review will be analyzed and presented as follows:   1. Description of studies:   Number of studies identified, number of studies that were eligible/ not eligible by location and date of publication, Location , Baseline characteristics of populations (including HIV positivity rate )   1. Description of study quality 2. Median TB incidences reported and summary estimates from meta-analysis (if number of studies permits). |
| ***Discussion*** | Strength of studies  Limitations of studies  Implications and recommendations for vaccine trials  Implications and recommendations for current policy and practice  Implications for further research |
