# Supplementary material for: Incidence of HIV-Associated Tuberculosis among Individuals Taking Combination Antiretroviral Therapy: A Systematic Review and Meta-Analysis
Source: PLoS One. 2014 Nov 13;9(11):e111209. doi: 10.1371/journal.pone.0111209 (PMC4230893; doi:10.1371/journal.pone.0111209)
Supplement: Table S3 — Data abstraction form. (DOCX) [file pone.0111209.s003.docx]

**TB Incidence on HAART Systematic Review- Data Abstraction form**

| Date of abstraction | |  | |
| --- | --- | --- | --- |
| Initials of abstractor | |  | |
| First Author | |  | |
| Date of publication | |  | |
| Location | |  | |
| World Bank Classification of country  *(high income /middle income/low income)* | |  | |
| Background HIV rates in country/ setting  (See UNAIDS estimates) | |  | |
| Background TB rates in the country  (high burden/ medium burden/ low burden)  See STOP TB classification | |  | |
| Objectives of study | |  | |
| Study design | |  | |
| Characteristics of study population at baseline | | \| Median/ mean age of participants  [#(IQR)] \|  \| \| --- \| --- \| \| % males \|  \| \| % current IPT \|  \| \| % past use of IPT \|  \| \| % with prior History of TB \|  \| \| Mode of transmission of HIV \|  \| \| % on ART \|  \| \| % ART naïve \|  \| \| Median/Mean CD4 count at study entry  [#(IQR)] \|  \| \| Median/Mean CD4 count at ART initiation  [#(IQR)] \|  \| \| ARVs drug regimens  [ PI based/ NNRTI based] \|  \| \| % on second line therapy \|  \| \| % on salvage therapy \|  \| | |
| TB case ascertainment  [ tick next to the appropriate box (es)] | | \| Defined as TB treatment started \|  \| \| --- \| --- \| \| Defined as definite or probable depending on clinical features and or smear microscopy or mycobacterium culture or genexpert \|  \| \| Defined as any TB cases reported/ notified \|  \| \| Self reported TB \|  \| | |
| Median/mean duration of follow up in months  [#, IQR] | |  | |
| Median/ Mean duration on cART  [#, IQR] | |  | |
| Sample size | |  | |
| Total person-years of follow up | |  | |
| # and % prevalent TB at  baseline | All TB | ------------- | _________% |
|  | PTB | ------------- | _________% |
|  | EPTB | ------------- | _________% |
| # and % incident TB cases | All TB | ------------- | _________% |
|  | PTB | ------------- | _________% |
|  | EPTB | ------------- | _________% |
| Characteristics of incident TB cases | | \| Median/mean age at TB diagnosis  [#, IQR)] \|  \| \| --- \| --- \| \| % IPT use at time of TB diagnosis \|  \| \| % previous IPT use \|  \| \| % previous TB \|  \| \| % male \|  \| \| % PTB \|  \| \| % extra pulmonary TB \|  \| \| Median /mean CD4 count at TB diagnosis  [#, IQR)] \|  \| | |
| Overall TB incidence rate in cohort | |  | |
| CD4 counts used for stratification of incidence  [Tick as appropriate] | | Current  At baseline  At cART initiation | |
| TB incidence rates reported in the paper stratified by CD4 count | | \| CD4 category (e.g. ….) \| # cases \| Person years \| TB incidence \| \| --- \| --- \| --- \| --- \| \| <100 \|  \|  \|  \| \| 101- 200 \|  \|  \|  \| \| 201-350 \|  \|  \|  \| \| 351-500 \|  \|  \|  \| \| >500 \|  \|  \|  \| | |
| TB incidences stratified by duration on cART | | \| **Duration** \| **# cases** \| **Person-years** \| **TB incidence** \| \| --- \| --- \| --- \| --- \| \| <3 months \|  \|  \|  \| \| 3-6 months \|  \|  \|  \| \| 6- 12 months \|  \|  \|  \| \| 12- 24 months \|  \|  \|  \| \| 24- 36 months \|  \|  \|  \| \| >36 months \|  \|  \|  \| | |
| TB incidence given TB history at initiation of cART | | \| **Prior history of TB at cART initiation** \| **# cases** \| **Person-years** \| **TB incidence** \| \| --- \| --- \| --- \| --- \| \| Yes \|  \|  \|  \| \| No \|  \|  \|  \| | |
| Is study eligible for inclusion in the review | |  | |
