# Supplementary material for: Incidence of HIV-Associated Tuberculosis among Individuals Taking Combination Antiretroviral Therapy: A Systematic Review and Meta-Analysis
Source: PLoS One. 2014 Nov 13;9(11):e111209. doi: 10.1371/journal.pone.0111209 (PMC4230893; doi:10.1371/journal.pone.0111209)
Supplement: Table S4 — Modified Ottawa Scale. (DOC) [file pone.0111209.s004.doc]

**Assessment of quality of a cohort study – Modified Newcastle Ottawa Scale**

**Author: Year: Country:**

| **Selection** (tick one or both boxes, as appropriate) |  |
| --- | --- |
| 1. Samplinga) The authors reported sampling method used ★b) The authors assessed and reported on sampling bias ★c) The authors did not report sampling method usedd) The authors did not assess and report on sampling bias | 🞏  🞏  🞏  🞏 |
| 2. Demonstration/evaluation that clients did not have TB at start of studya) Yes ★b) No | 🞏  🞏 |
| **Outcome** (tick one box in each section) |  |
| 1. Assessment of outcomea) Lab confirmation – microscopy or culture ★c) Self report d) clinical signs and symptoms only/ no description | 🞏  🞏  🞏 |
| 2. Was follow up long enough for outcomes to occura) Yes, if median duration of follow-up >= 9 months ★b) No, if median duration of follow-up < 9 months | 🞏  🞏 |
| 3. Adequacy of follow up of cohortsa) Complete follow up: all subjects accounted for ★b) Subjects lost to follow up unlikely to introduce bias (<=20%) ★ or description of those lost suggesting no different from those followedc) Subjects lost, likely to introduce bias (<80%) or description of lost suggest they are different from those followedd) No statement | 🞏  🞏  🞏  🞏 |

**Notes:**

This scale has been modified to suit study purposes. The number of stars awarded to each study will be tabulated. However, in the manuscript, quality assessment findings will be purely descriptive. Quality assessments will also be essential in guiding the inclusion or exclusion of studies in sensitivity analyses.

**Box 1: Criteria used to assess study quality**

1. Sampling method used

## The authors reported sampling method used (score 1)

## The authors assessed and reported on sampling bias (score 1)

## The authors did not report sampling method used

The authors did not assess and report on sampling bias

1. Screening of TB at cohort entry

Author reported/made a statement on the exclusion of participants with TB or on TB treatment (score 1)

1. Assessment of outcome

Laboratory confirmed (score 1)

- - 1. Smear microscopy (ZN or fluorescence microscopy)
    2. Culture – liquid or solid culture

Clinical only – TB associated symptoms, chest X-ray, starting TB treatment, response to anti-tuberculosis therapy

Not specified – none of the methods a-e or others specified

1. Median follow-up time

Median duration of follow up ≥9 months (score 1)

Median duration of follow up < 9 months

1. Adequacy of follow up of cohorts

## Complete follow up: all subjects accounted for (score 1)

## Subjects lost to follow up unlikely to introduce bias (<=20%) or description of those lost suggesting no different from those followed (score 1)

## Subjects lost, likely to introduce bias (<80%) or description of lost suggest they are different from those followed

No statement
